# Bleomycin-Induced Flagellate Erythema in a Patient Diagnosed with Ovarian Yolk Sac Tumor

**DOI:** 10.1155/2015/574708

**Published:** 2015-12-21

**Authors:** Stergios Boussios, Michele Moschetta, Jennifer McLachlan, Susana Banerjee

**Affiliations:** Gynaecology Unit, The Royal Marsden NHS Foundation Trust, Sycamore House, Downs Road, Sutton, Surrey SM2 5PT, UK

## Abstract

Flagellate linear hyperpigmentation can rarely be caused by the chemotherapy agent, bleomycin. Herein, we describe the case of a 20-year-old woman treated with bleomycin for an ovarian yolk sac tumor and review the prominent features of this form of dermatitis.

## 1. Introduction

Bleomycin is an antibiotic derived from* Streptomyces verticillus*. In low doses, the antineoplastic properties are achieved by inhibition of mitosis and at higher concentrations it blocks DNA uptake of thymidine in the S-phase of the cell cycle [1].

Bleomycin-induced toxicities are more pronounced in lung tissue and skin due to low concentrations of the metabolizing enzyme-bleomycin hydrolase in these organs. Numerous dermatological toxicities have been reported, such as alopecia, palmar plantar erythema, eczematous changes, sclerodermoid lesions, nail-bed changes, Raynaud's phenomenon, and digital gangrene [2].

## 2. Material

We report the case of a 20-year-old woman with no significant past medical or surgical history. An ultrasound examination demonstrated a suspicious ovarian cyst in pregnancy and the patient underwent fertility-preserving ovarian cancer resection with left oophorectomy. Routine pathological examination revealed replacement of the cyst by yolk sac tumor with a minor component of mature cystic teratoma.

**Figure 1:**
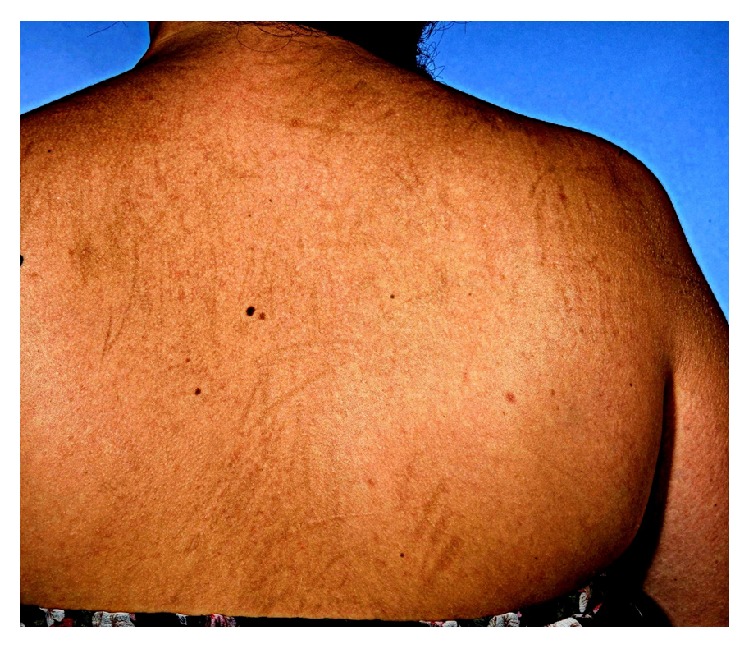
Fading flagellate dermatosis due to bleomycin.

The postsurgical recovery was uneventful and the postoperative CT scan identified lung metastases and extensive peritoneal disease. The alpha-fetoprotein (AFP) before the initiation of the adjuvant chemotherapy was noted to be 2,497 kU/L. The patient was treated with 4 cycles of 5-day schedule BEP (bleomycin 30,000 IU days 1, 8, and 15, etoposide 100 mg/m^2^ days 1–5, and cisplatin 20 mg/m^2^ days 1–5). Bleomycin was omitted after 3 cycles to reduce the risk of pulmonary fibrosis. The AFP level normalized by the end of the treatment and complete remission was achieved. Four months following chemotherapy, the patient noticed a dark streaked rash affecting her chest and the upper part of the dorsum (Figure 1). Dermatological examination revealed an extensive violaceous and erythematous excoriated rash with a striking linear configuration and a postinflammatory hyperpigmentation. No treatment modality was prescribed and the lesions resolved completely 6 months after the cessation of the bleomycin. The patient remains disease free two years after the initial diagnosis.

## 3. Discussion

Flagellate erythema is recognised cutaneous toxicity of bleomycin first described in 1970 [3]. Subsequent studies revealed an incidence rate of 8% to 20% independently of the route of administration or type of malignant disease being treated [1]. It typically presents with itching, coinciding with the appearance of erythematous linear streaks which are found most commonly on the upper chest and back, limbs, and flanks.

A number of theories have been postulated in the pathogenesis of flagellate erythema. Microtrauma such as abrasions or pressure on areas of bony prominences has been described, but the exact mechanisms remain to be determined [4]. One theory is that the linear lesions could be induced by rubbing or scratching which induces subclinical local vasodilatation resulting in an excessive in situ accumulation of bleomycin [5]. It is generally pruriginous and may have the likeness of postinflammatory hyperpigmentation from the beginning or start as erythematous urticaria-like lesions which progress to residual hyperpigmentation. As the rash becomes less erythematous, the affected areas usually become deeply pigmented. An additional proposed mechanism for bleomycin-induced flagellate hyperpigmentation includes stimulation of melanocytes by adrenocorticotrophic hormone [6].

The reaction was originally considered dose-dependent; however, it usually occurs after cumulative doses of 100–300 mg. It has also been reported after small doses, and there is a case described in the literature of flagellate hyperpigmentation after an intralesional injection of 14 units of bleomycin for plantar warts [7]. The interval between the administration of drug and the onset of flagellate erythema ranges variably and can occur anywhere from the first day to 9 weeks after bleomycin administration and can last up to six months [2]. Physical examination usually reveals linear intermingled streaks formed by rows of adjoining firm papules, which can show evidence of punctuate hemorrhages or can be pustular in character. Histological and ultrastructural studies indicated that bleomycin reduced the epidermal turnover, resulting in a prolonged contact between melanocytes and keratinocytes. Polla et al. found spongiosis mainly localised in the basal layer of the epidermis together with a perivascular infiltrate consisting of lymphocytes and neutrophil granulocytes as well as pigmentary incontinence [8].

The eruptions are usually self-limiting and usually subside 3-4 months later after the cessation of bleomycin [9]; however, persistence of hyperpigmented streaks for up to 1 year after treatment has been reported [7]. Most cases are reversible following discontinuation of the agent. Antihistamines and topical and oral corticosteroid use have been described and can reduce pruritus [10].

## 4. Conclusion

Clinicians should be aware of this rare cutaneous manifestation associated with bleomycin and counsel patients appropriately regarding the reversibility of the condition.

## References

[1] Lee H.-Y., Lim K.-H., Ryu Y., Song S.-Y. (2014). Bleomycin-induced flagellate erythema: a case report and review of the literature. *Oncology Letters*.

[2] Chen Y.-B., Rahemtullah A., Breeden E., Hochberg E. P. (2007). Bleomycin-induced flagellate erythema. *Journal of Clinical Oncology*.

[3] Moulin G., Fière B., Beyvin A. (1970). Cutaneous pigmentation caused by bleomycin. *Bulletin de la Societe Francaise de Dermatologie et de Syphiligraphie*.

[4] Yamamoto T., Nishioka K. (2006). Flagellate erythema. *International Journal of Dermatology*.

[5] Biswas A., Chaudhari P. B., Sharma P., Singh L., Julka P. K., Sethuraman G. (2013). Bleomycin induced flagellate erythema: revisiting a unique complication. *Journal of Cancer Research and Therapeutics*.

[6] Cortina P., Garrido J. A., Tomas J. F., Unamuno P., Arnijo M. (1990). ‘Flagellate’ erythema from bleomycin. With histopathological findings suggestive of inflammatory oncotaxis. *Dermatologica*.

[7] Abess A., Keel D. M., Graham B. S. (2003). Flagellate hyperpigmentation following intralesional bleomycin treatment of verruca plantaris. *Archives of Dermatology*.

[8] Polla L., Mérot Y., Slosman D., Polla B., Olgiati D., Saurat J. H. (1985). Linear pigmentogenic dermitis after scintigraphy using bleomycin. *Annales de Dermatologie et de Vénéréologie*.

[9] Todkill D., Taibjee S., Borg A., Gee B. C. (2008). Flagellate erythema due to bleomycin. *British Journal of Haematology*.

[10] Mota G. D., Penna A. M., Soares R. C., Baiocchi O. C. (2014). Bleomycin-induced flagellate dermatitis. *Revista Brasileira de Hematologia e Hemoterapia*.

